# Automated Fractal Analysis of Right and Left Condyles on Digital Panoramic Images Among Patients With Temporomandibular Disorder (TMD) and Use of Machine Learning Algorithms in the Diagnosis of TMD

**DOI:** 10.7759/cureus.100165

**Published:** 2025-12-27

**Authors:** Sumeet N Raut, Prashant B Patil

**Affiliations:** 1 Oral Medicine and Radiology, Employees' State Insurance Corporation Dental College and Hospital, Kalaburagi, IND

**Keywords:** automated fractal analysis, confusion matrix, fractal analysis, fractal dimension, imagej, machine learning, orthopantomogram, receiver operating characteristic curve, temporomandibular disorder, xgboost

## Abstract

Background & purpose

The diagnosis of temporomandibular disorder (TMD) can be a challenging and arduous task. The role of advanced imaging modalities, such as computed tomography & magnetic resonance imaging, in the evaluation of temporomandibular joints is indispensable. However, panoramic imaging remains a widely available and cost-effective imaging modality in many clinical setups. This study aims to automate fractal analysis of mandibular condyles on panoramic radiographs using ImageJ macros code and to compare fractal dimensions between TMD patients and age-group-matched healthy controls. Additionally, the study evaluates age- and gender-related variations in fractal dimensions and investigates the utility of machine learning classifiers for differentiating patients with TMD from healthy individuals.

Materials and methods

A total of 220 subjects were selected for the study, with 110 patients with TMD & 110 healthy controls. Additionally, they were divided into four age groups: 18-29 years, 30-39 years, 40-49 years, and ≥ 50 years. Right & left condyles on 220 digital orthopantomogram (OPG), of which 110 belonged to patients with TMD and 110 to age group-matched healthy controls, were analyzed with ImageJ 1.42q for the automated calculations of fractal dimensions (FD) with the box counting method. Data obtained was utilized to train (70%), validate (15%), and test (15%) six machine learning (ML) algorithms: random forest classifier, logistic regression, support vector machine (SVM), gradient boosting, K-Nearest Neighbors & XGBoost to differentiate between patients with TMD & healthy controls based on the features such as FD values and age & gender variables.

Results

For right & left condyles, the FD distribution showed a similar pattern across most of the age groups, except for the control group between the ages of 18 and 29 years. FD values for the patients with TMD were statistically lower in most of the age groups compared to those of the controls, with the greatest visual differences observed in the 30-39 age group and the female participants. Overall mean FD value for patients was 1.2232 and controls was 1.2944. For ML, the best performing model was XGBoost with the validation F1 Score: 0.9620, test F1 Score: 0.8750, test accuracy: 0.8936, test precision: 0.8750, test recall: 0.8750, and test receiver operating characteristic area under the curve (ROC AUC): 0.9449 for discriminating between patients with TMD & healthy controls.

Conclusion

Patients with TMD showed highly statistically lowered FD values for the mandibular condyles compared to those of the age group-matched controls, with significant overall and group-wise gender differences. FD values show robust prognostic power for differentiating patients with TMD from the respective controls. The application of a ML algorithm, i.e., the XGBoost, attained an ROC-AUC of 0.9449, indicating excellent diagnostic performance, which validates the capability of FD as a useful diagnostic radiographic marker.

## Introduction

Temporomandibular disorders (TMD) constitute a spectrum of musculoskeletal disorders, presenting clinically as pain, dysfunction, joint sounds, masticatory muscle tenderness, and radiographically present as degenerative changes of the mandibular condyle & articular eminence. Roughly 5-12% of the general population accounts for TMD, with a bimodal peak at 21 and 53 years of age, with a female-to-male ratio of 3:1 [1]. Alterations in the bony outline of the condyle & articular eminence due to different causative factors such as occlusion, parafunctional habits, and deep pain inputs may result in degenerative joint diseases of the temporomandibular joint (TMJ), particularly osteoarthritis [2]. Imaging modalities, for instance, panoramic radiography and cone-beam computed tomography (CBCT) yield valuable structural information but are likely to entirely overlook the microstructural bone changes in relation to TMD [3]. Studies comparing panoramic radiography with CBCT have demonstrated differences in trabecular fractal dimension (FD) metrics between the two modalities and indicate that they are not directly interchangeable for bone structure analysis, while panoramic radiographs remain widely used due to lower radiation exposure and clinical accessibility [4,5]. Although CBCT offers three-dimensional information, panoramic imaging provides a practical, routinely acquired dataset for FD analysis in observational studies.

Recent advances in imaging analysis, principally fractal analysis (FA), provide a quantifiable technique to assess the trabecular bone structure of TMJ and therefore allow for the objective assessment of degenerative changes and bone remodeling [6,7]. FA is a mathematical means of quantifying complexity and irregularity present in bone microstructure, with FD being a principal marker of trabecular complexity. The lower the FD, the more bone resorption and decrease in density is usually implicated, and a rise in FD reflects a denser, more complex trabecular structure [8,9]. Previous studies have indicated that patients with TMD have a lower FD of the mandibular condyle, indicating continuous bone remodeling and degenerative processes [10]. Additionally, FA has been employed to evaluate asymmetry between the right and left condyles, which may potentially provide a measure of the severity and progression of TMD [11].

Although valuable, manual FA measures are time-consuming and operator-dependent, and the involvement of multiple steps may constrain their clinical use & reproducibility. Automated FA with the ImageJ software (Rasband, W. S. (1997-2018). ImageJ (Version 1.42q). U. S. National Institutes of Health, Bethesda, Maryland, USA. Available from https://imagej.net/ij/.), an open-source image processing program, provides a reproducible and standardized technique for the assessment of trabecular complexity in TMJ radiographs [12]. The box-counting algorithm of ImageJ facilitates fast, objective, and reproducible calculation of FD, thereby evading human error and augmenting the efficiency of fractal-based diagnostics [13].

To the best of our knowledge, this is a unique study wherein automated FA has been used to train six different machine learning (ML) algorithms to differentiate between patients with TMD & healthy controls. The primary objective of the present study is to generate & compare the automated FA of mandibular condyles with ImageJ macros code of patients with TMD and their age-group matched healthy controls. The secondary objectives of the study are to explore age-based and gender-based differences in the FD & to develop ML classifiers. After obtaining the automated FD, six different ML algorithms: random forest classifier, logistic regression, support vector machine (SVM), gradient boosting, K-Nearest Neighbors & XGBoost were trained, validated & tested for different features to differentiate between patients with TMD & healthy controls. Using automated FA, this research intends to enhance the objectivity and clinical utility of FA in TMD diagnosis, potentially enabling early diagnosis and treatment planning in the clinical settings lacking advanced diagnostic modalities such as CBCT & MRI.

## Materials and methods

Study design

**Figure 1:**
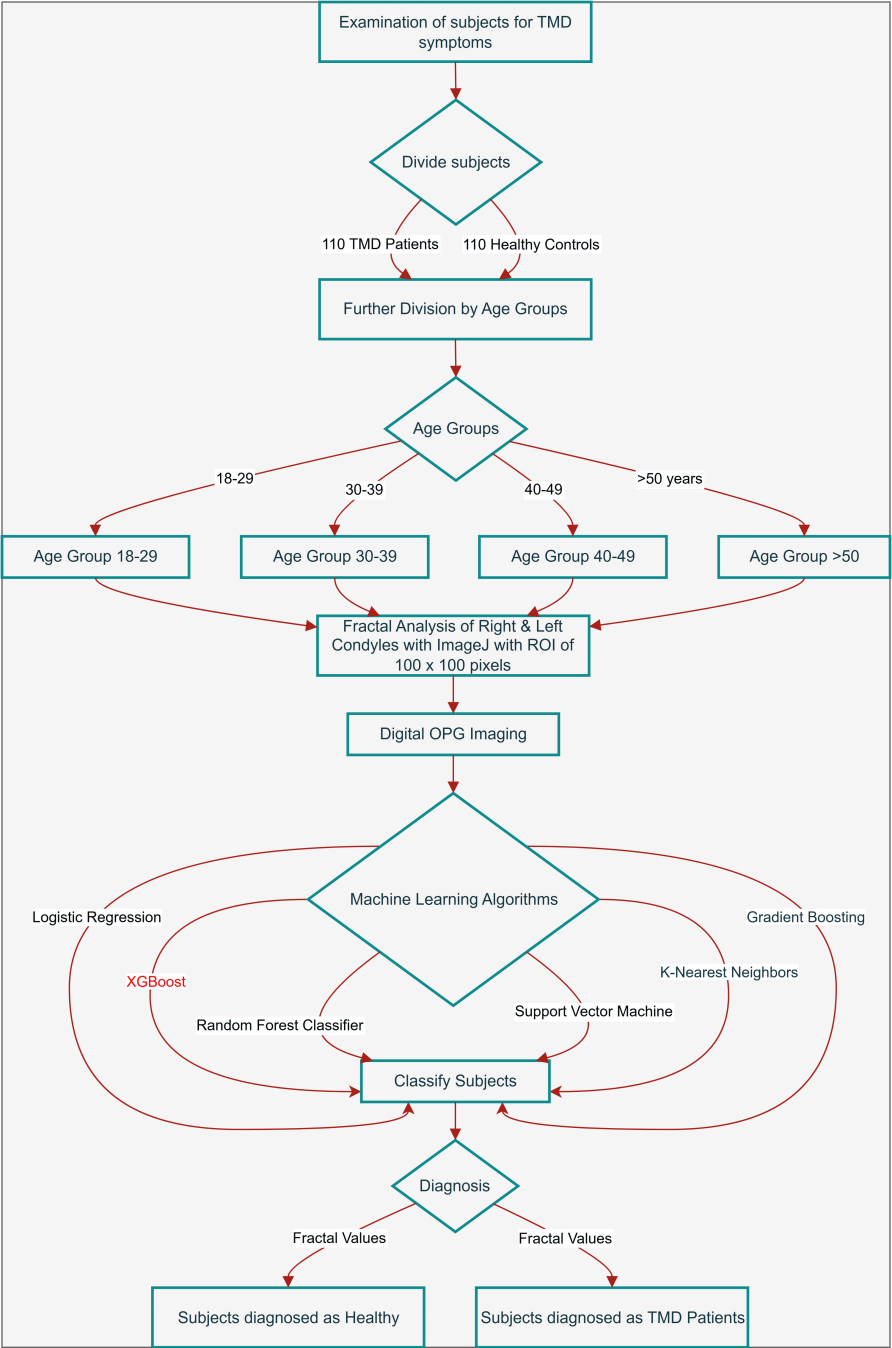
Machine learning study design TMD: Temporomandibular disorders; ROI: Region of interest; OPG: orthopantomogram. Image credit: Image created by Dr. Sumeet Raut using draw.io (Wiesbaden, Germany).

This was a cross-sectional, observational study conducted in the Department of Oral Medicine and Radiology of the Employees' State Insurance Corporation Dental College and Hospital, Kalaburagi, Karnataka, India, from June 2023 to April 2025. The participants of the study were patients with TMD & their age group-matched controls aged between 18 and 75 years. Ethical approval was obtained from the Institutional Review Board (IRB) before conducting the study (Approval No. 532/GLBDC/IEC/RP/2023/11). Sample size was determined at a 95% confidence level, error margin of 5%, and a population proportion of 18%. For an equal number of cases and controls, the sample size was fixed at 220, consisting of 110 patients with TMD and 110 age group-matched healthy controls. Further, they were divided into four age groups: 18-29 years, 30-39 years, 40-49 years, and ≥50 years. Further study design was developed as shown in Figure 1. 

Inclusion criteria included patients with pain and dysfunction of the TMJ & patients willing to be part of the study and providing informed consent. Exclusion criteria included autoimmune, neurologic, endocrine, or metabolic bone diseases; history of head and neck radiation therapy; history of prior TMJ surgery or jaw trauma; and pregnant women. Diagnostic criteria for Temporomandibular Disorders (DC/TMD) was used for establishing diagnosis of patients with TMD & radiographic findings such as osteophytes, sclerosis, flattening, erosion, and subcondylar cysts (Ely's cysts) were used to confirm the diagnosis [14]. Patients with TMD were diagnosed on the basis of the clinical characteristics of pre-auricular tenderness, reduced inter-incisal distance, clicking or crepitus, masticatory muscle tenderness, any of these with deviation, deflection, or malocclusion. In the control group, there was abstinence from pain, dysfunction, and history of craniofacial trauma. Panoramic radiographs were acquired using the PaX-I Panoramic X-ray Machine (VATECH, India) with the settings of Tube voltage: 70 kVp, Tube current: 4 mA, Exposure time: 13.5 sec for HD mode, Focal spot size: 0.5mm, which generated digital images with a resolution of 240 dpi.

Automated fractal analysis (FA)

A 100 × 100-pixel region of interest (ROI) was delineated over the right and left mandibular condyles. ROIs were standardized for radiographic change, including osteophytes, sclerosis, flattening, erosion, and subcondylar cysts (Ely's cysts). A measurement of 100 × 100 pixels was adopted to allow enough coverage of the subchondral bone decreasing the possibility of overlapping anatomical structures, and to allow FA to be trusted.

**Figure 2:**
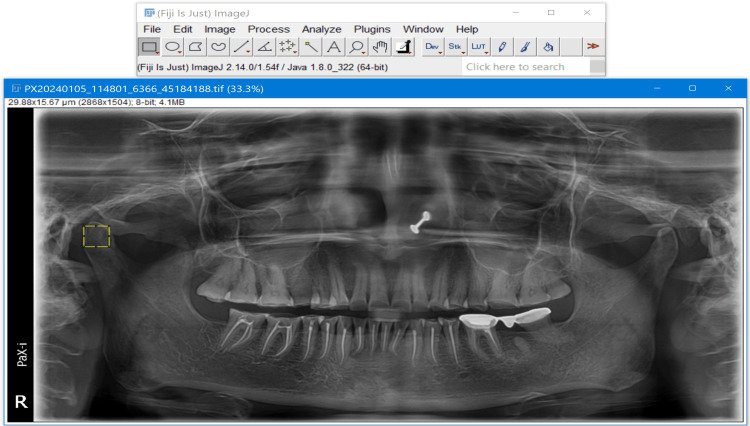
Region of interest (ROI) selection of 100x100 pixels of condyles on a digital panoramic image with the ImageJ software

FA was performed with ImageJ version 1.42q using the box-counting algorithm of Rudolf & White (Figure 2) [15].

The automation code created for this analysis is available online at a GitHub repository (Appendix A).

After selection of ROI the (x,y) parameters in the ImageJ macros code was adapted & the process was run for every subject, the 110 patients with TMD & 110 healthy age group-matched controls twice, i.e. right & left condyles separately, resulting in a total of 440 data sets. The complete ImageJ macro script, ROI placement protocol, and ML hyperparameters are available at a GitHub repository (Appendix B).

**Figure 3:**
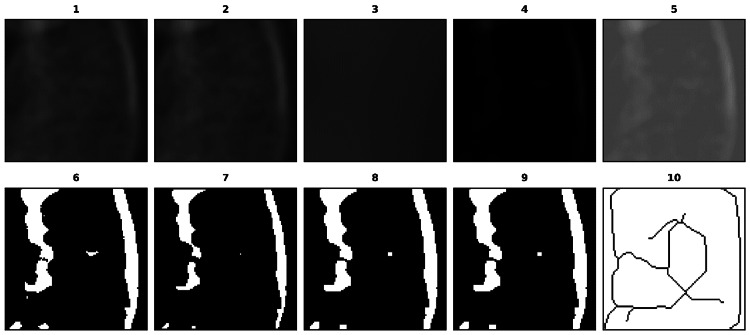
Montage image of steps involved in the automated fractal analysis (FA) (1) Selected region of interest (ROI); (2) Duplicated ROI; (3) After applying the Gaussian filter; (4) Subtraction of image (3) from (2); (5) Addition of 128 pixels; (6) Binarization; (7) Erosion; (8) Dilation; (9) Inverted version; and (10) Skeletonization.

The ten steps involved in the FA analysis are shown in Figure 3.

Observer reliability for ROI selection

Two experienced senior examiners (10 and five years in Oral Medicine & Radiology) independently analyzed the OPGs. The two examiners read the images twice, two weeks apart, to eliminate intra- and inter-examiner variation. An intraclass correlation coefficient (ICC (2,1)) was used to estimate absolute agreement between the two examiners. To enhance reproducibility, all image preprocessing parameters, ROI placement criteria, and the complete ImageJ macro used for automated FD analysis are reported online (Appendix B), in line with recommended practices for quantitative imaging studies [16]. The conventional method of FD calculations involves application of these steps manually from the drop-down menu in ImageJ. In order to standardize the process and prevent operator bias, the box-counting method was automated by generating code in ImageJ's built-in macros to enable the calculation of FD in a standard and reproducible manner.

Machine learning (ML) data preparation

ML model configuration, including hyperparameters, cross-validation strategy, and fixed random seed values, was explicitly reported to support reproducibility and transparency, consistent with recommended reporting standards for clinical machine learning studies [17].

FA was performed for a total of 220 individuals with 220 FD values for each condyle, generating a total of 440 FD values. An aggregate set of 621 parameters comprising different features was used to train six ML algorithms, out of which the training set involved 433 parameters, and the validation & test sets consisted of 94 parameters each. Features such as age, gender, left condyle FD (LC_FD), and right condyle FD (RC_FD) were standardized using StandardScaler. Six different machine learning algorithms: random forest classifier, logistic regression, SVM, gradient boosting, K-Nearest Neighbors & XGBoost were employed to differentiate between patients with TMD & healthy controls.

ML hyperparameters and random seeds

Six ML models were trained to classify patients with TMD vs. healthy controls. Where hyperparameters were not explicitly set in the original code, default values from scikit-learn 1.3.0 and XGBoost 1.7.5 were applied. Random seeds were set retrospectively to 42 to ensure reproducibility. Model performance metrics (accuracy, precision, recall, F1-score, receiver operating characteristic area under the curve (ROC-AUC)) were calculated using five-fold cross-validation (Appendix C).

Statistical analysis

Statistical analysis comprised calculation of descriptive statistics (frequency, percentage, mean, and standard deviation) for the right & left condyles of patients & age group-matched controls. The Shapiro-Wilk test was applied to confirm data normality, independent t-test was used to compare patients with TMD and controls statistically. A p-value of <0.05 was considered statistically significant.

## Results

FA of right & left condyles

**Figure 4:**
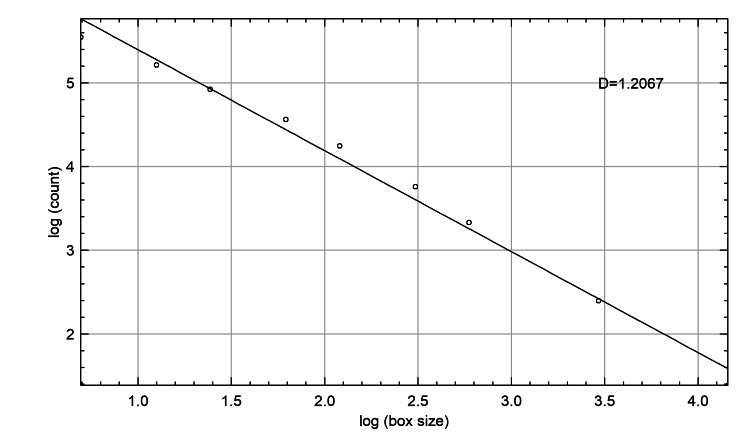
Output graph generated after carrying out automated fractal analysis using the box-counting method

FA on OPG was employed to compare the trabecular bone structure of the mandibular condyles in patients with TMD with that of age group-matched healthy controls. Interobserver agreement for ROI selection was high, with an ICC (2,1) of 0.87 (95% CI: 0.81-0.92), indicating good reliability. FD measures were taken for the left condyle (LC_FD) and right condyle (RC_FD), and the subjects were divided into four groups according to age (18-29, 30-39, 40-49, and ≥50 years). The study population included 112 male subjects (50.9%) and 108 female subjects (49.1%), with no significant demographic differences among patient and control groups (p>0.05), confirming comparable groups for analysis [18]. In addition to the conventional statistical analysis, ML classifier models were also developed using the FD measures as prognosticators. Figure 4 shows the FD value generated after automated FA.

Table 1 shows the descriptive statistics for the patients' LC_FD & RC_FD.

**Table 1:** Descriptive statistics of the left condyle fractal dimension (LC_FD) & right condyle fractal dimension (RC_FD) in the study population Male patients showed the highest value for mean LC_FD of 1.2539 in the age group 18-29 years & lowest value of 1.2150 in ≥50 age group. In the case of female patients, the highest & lowest value of 1.2339 & 1.1857 for mean LC_FD was recorded in the age group of 30-39 years & 18-29 years, respectively. For RC_FD, male patients showed the highest value for mean of 1.2675 in the age group 18-29 years & lowest value of 1.182 in 40-49 years age group. In female patients, the highest & lowest value for mean RC_FD was 1.2406 & 1.192, and was recorded in the age group of 30-39 years & 40-49 years, respectively.

Age group (years)	Number of male patients	Number of female patients	Total number of patients	Mean LC_FD for male patients	Standard deviation of LC_FD for male patients	Mean LC_FD for female patients	Standard deviation of LC_FD for female patients	Mean LC_FD for overall patient population	Standard deviation of LC-FD for overall patient population
18-29	12	12	24	1.2539	0.0909	1.1857	0.1066	1.2198	0.103
30-39	7	26	33	1.251	0.0511	1.2339	0.0758	1.2376	0.0709
40-49	13	13	26	1.2254	0.0911	1.2122	0.0722	1.2188	0.0808
≥50	15	12	27	1.215	0.0934	1.2103	0.07	1.2129	0.0823
	Number of male patients	Number of female patients	Total number of patients	Mean RC_FD for male patients	Standard deviation of RC_FD for male patients	Mean RC_FD for female patients	Standard deviation of RC_FD for female patients	Mean RC_FD for overall patient population	Standard deviation RC_FD for overall patient population
18-29	12	12	24	1.2675	0.0858	1.2367	0.0914	1.2521	0.0881
30-39	7	26	33	1.252	0.0674	1.2406	0.0821	1.243	0.0783
40-49	13	13	26	1.182	0.0986	1.192	0.0788	1.187	0.0876
≥50	15	12	27	1.2392	0.0682	1.1938	0.0852	1.2191	0.0781

Table 2 shows descriptive statistics for age group-matched healthy controls for LC_FD & RC_FD.

**Table 2:** Descriptive statistics of left condyle and right condyle fractal dimensions (LC_FD and RC_FD) in the age-matched control groups Male controls showed the highest & lowest values of mean LC_FD values of 1.3008 & 1.2497 in the age group of 18-29 & ≥50 years, respectively. Female controls recorded the highest value of 1.3462 in the 18-29 year age group & lowest value of 1.285 in the 40-49 years age group. Male controls showed the highest & lowest values of mean RC_FD of 1.3212 & 1.2392 in the age group of 40-49 & 30-39 years, respectively, while female controls recorded the highest value of 1.3265 in the 40-49 year age group & lowest value of 1.2361 in the ≥50 years age group.

Age group (years)	Number of male control patients	Number of female control patients	Total number of controls	Mean LC_FD in male controls	Standard deviation of LC_FD in male controls	Mean LC_FD in female controls	Standard deviation of LC_FD in female controls	Mean LC_FD in overall control population	Standard deviation of LC_FD in overall control population
18-29	13	11	24	1.3008	0.0645	1.3462	0.0434	1.3216	0.0594
30-39	12	21	33	1.2893	0.0847	1.3028	0.086	1.2979	0.0844
40-49	14	12	26	1.2899	0.073	1.285	0.083	1.2877	0.0762
≥50	15	12	27	1.2497	0.0562	1.3007	0.0634	1.2724	0.0638
	Number of male control patients	Number of female control patients	Total number of controls	Mean RC_FD in male controls	Standard deviation of RC_FD in male controls	Mean RC_LD in female controls	Standard deviation of RC_FD in female controls	Mean RC_FD in overall control population	Standard deviation of RC_FD in overall control population
18-29	12	12	24	1.2412	0.1455	1.3004	0.1103	1.2708	0.1298
30-39	17	16	33	1.2392	0.0958	1.3095	0.0963	1.2733	0.101
40-49	10	16	26	1.3212	0.1025	1.3265	0.0727	1.3245	0.0834
≥50	15	12	27	1.2669	0.0757	1.2361	0.1106	1.2532	0.0922

Table 3 shows the normality distribution test results for the RC_FD & LC_FD of patients with TMD & controls according to the different age groups.

**Table 3:** Shapiro-Wilk test results for the distribution of the fractal dimension values for the left (LC_FD) & right (RC_FD) condyles in the different age groups The Shapiro-Wilk test for LC_FD & RC_FD was true for all age groups except for the RC_FD of controls in the age group of 18-29 years, suggesting that this group followed a non-normal distribution of data.

Group	Age group (years)	Shapiro-Wilk test for LC_FD	p-value for LC_FD	Normality distribution test results for LC_FD	Shapiro-Wilk test for RC_FD	p-value for RC_FD	Normality distribution test results for RC_FD
Control	18-29	0.978835	0.873467	TRUE	0.911479	0.037979	FALSE
	30-39	0.969389	0.463511	TRUE	0.938782	0.062617	TRUE
	40-49	0.923375	0.053975	TRUE	0.97009	0.625648	TRUE
	≥50	0.988685	0.988122	TRUE	0.951778	0.236718	TRUE
Patient	18-29	0.920156	0.058875	TRUE	0.958143	0.402255	TRUE
	30-39	0.969331	0.461943	TRUE	0.984007	0.895932	TRUE
	40-49	0.957456	0.343964	TRUE	0.963513	0.465362	TRUE
	≥50	0.964058	0.454946	TRUE	0.938355	0.110975	TRUE

**Figure 5:**
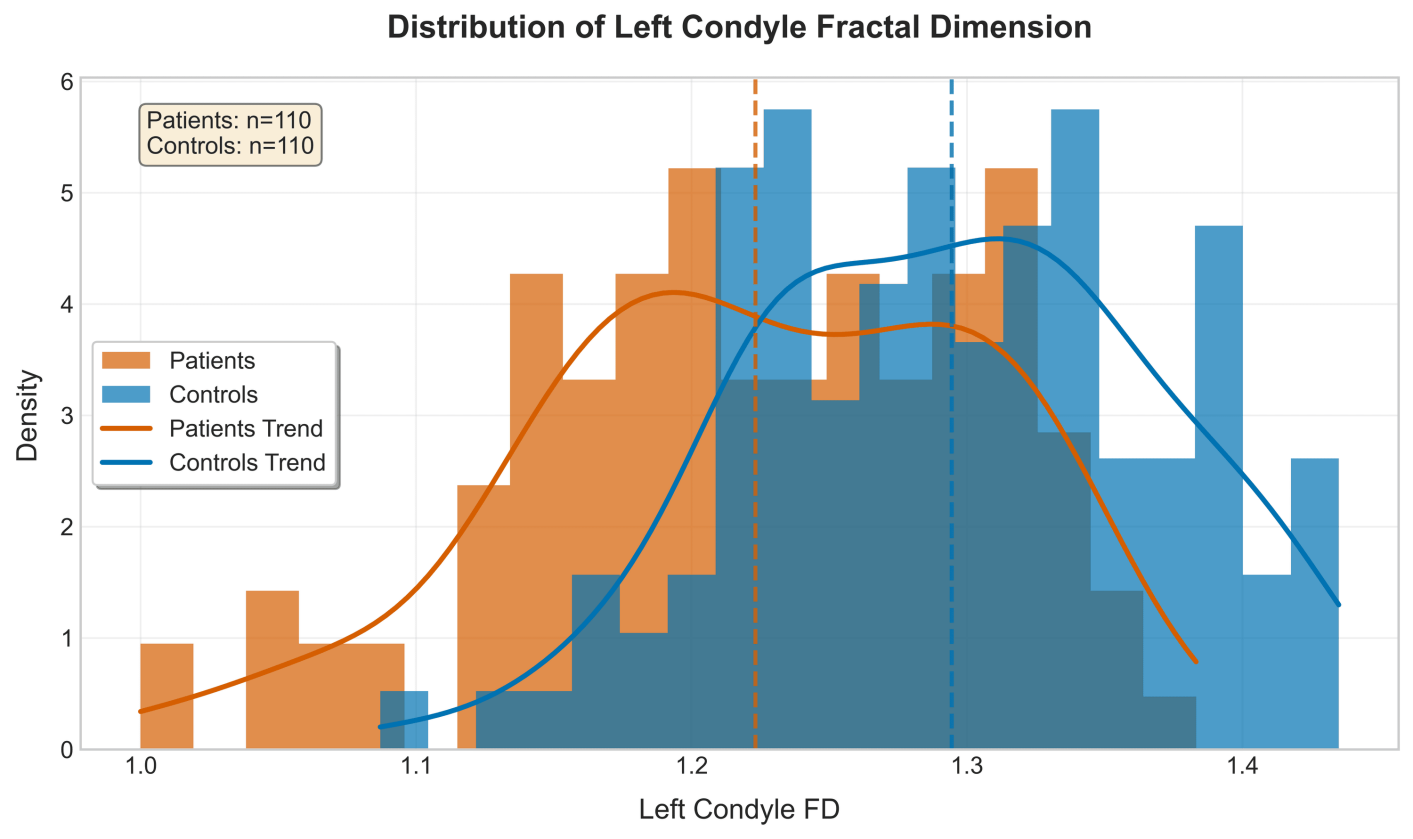
Histograms showing the distribution of fractal dimension (FD) for the left condyle of patients vs. controls

**Figure 6:**
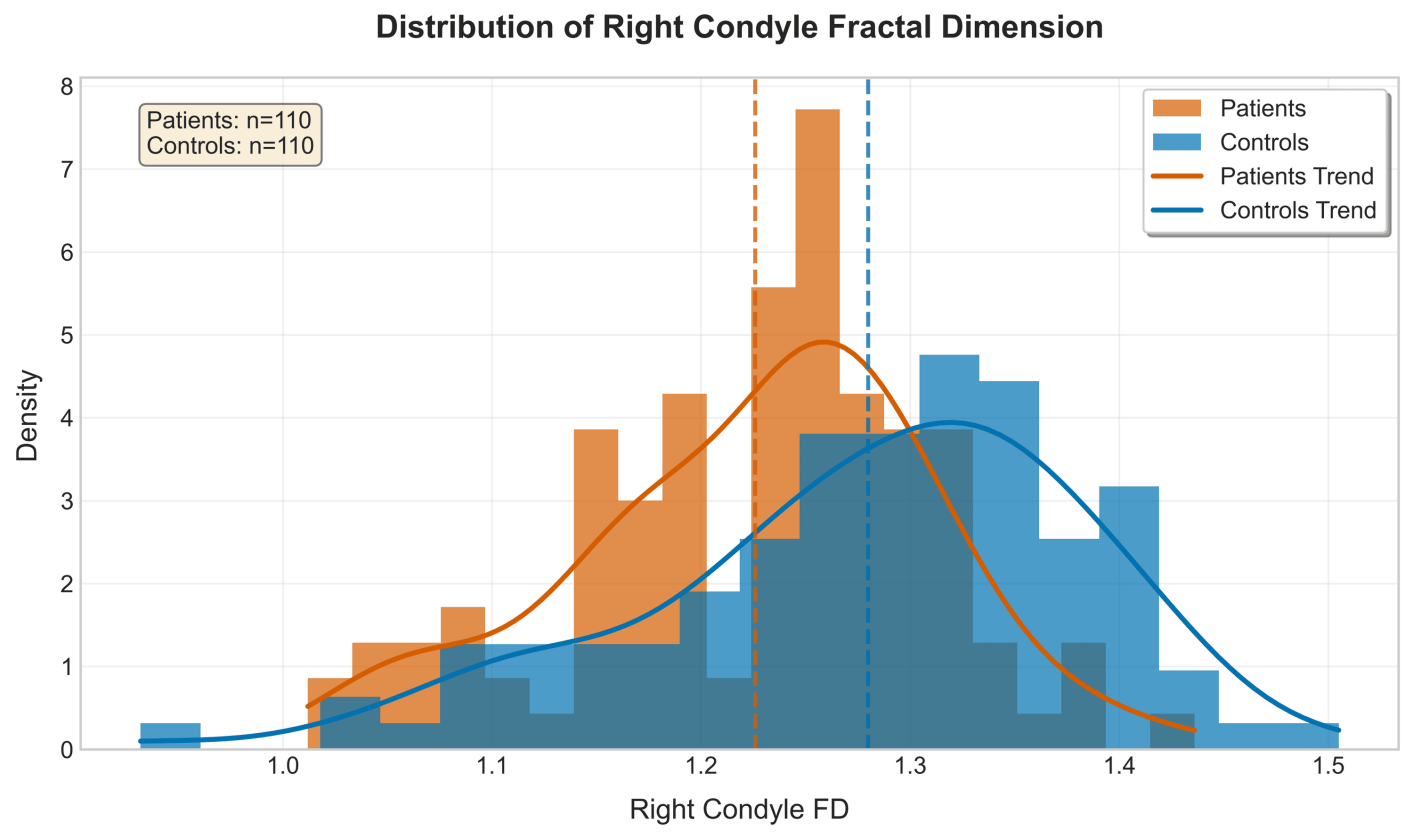
Histograms showing the distribution of fractal dimension (FD) for the right condyle of patients vs. controls

From the distribution pattern of RC_FD and LC_FD values, it can be interpreted that both patient & control groups showed normal distribution with mild skewness, & controls in general showed higher FD values compared to patients with TMD (Figures 5, 6).

**Figure 7:**
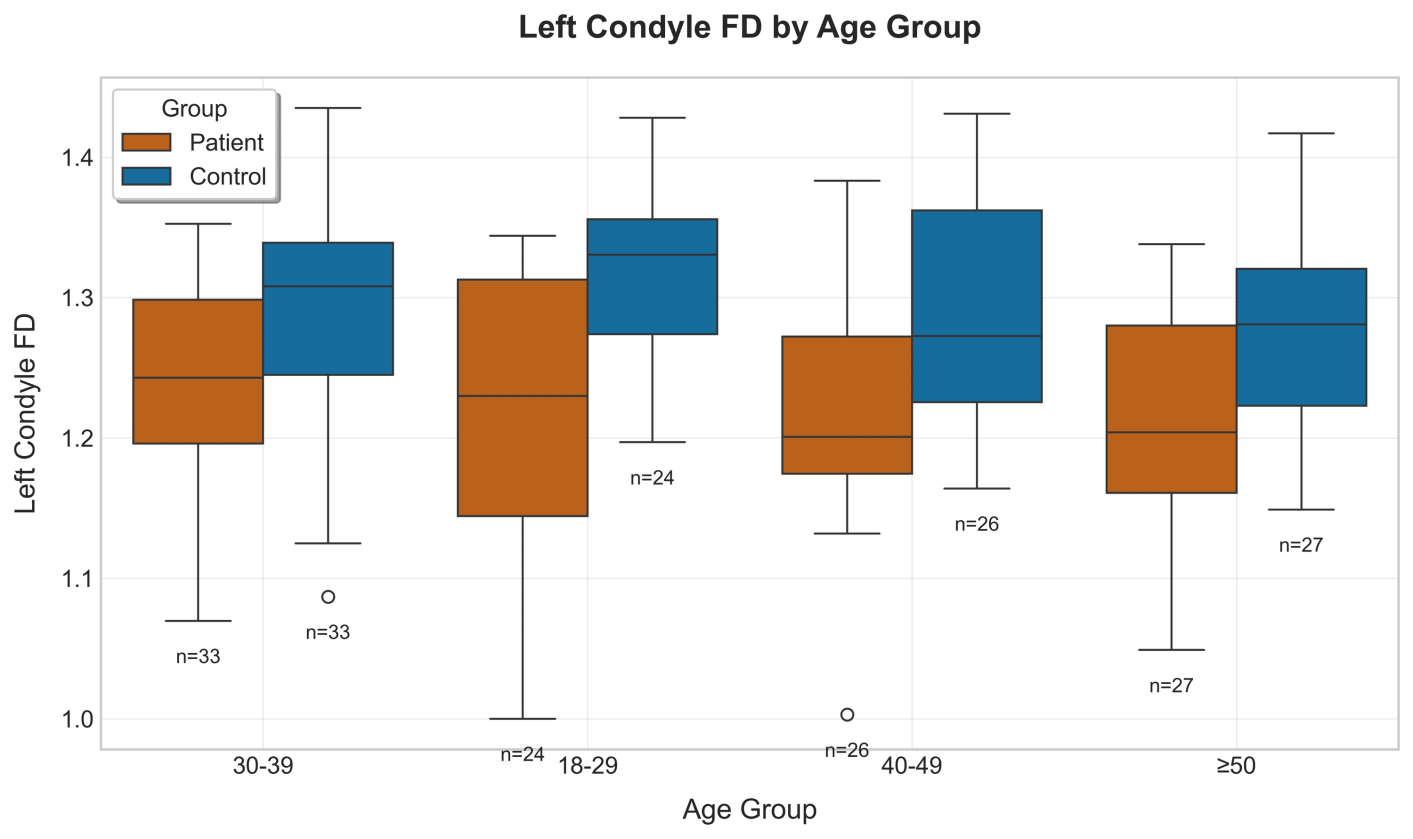
Boxplot showing the left condyle distribution of the patients & controls according to their age groups

Younger age groups (18-29) presented higher FD values, suggesting more complex bone structure & controls consistently showed higher median values across all the age groups (Figure 7).

**Figure 8:**
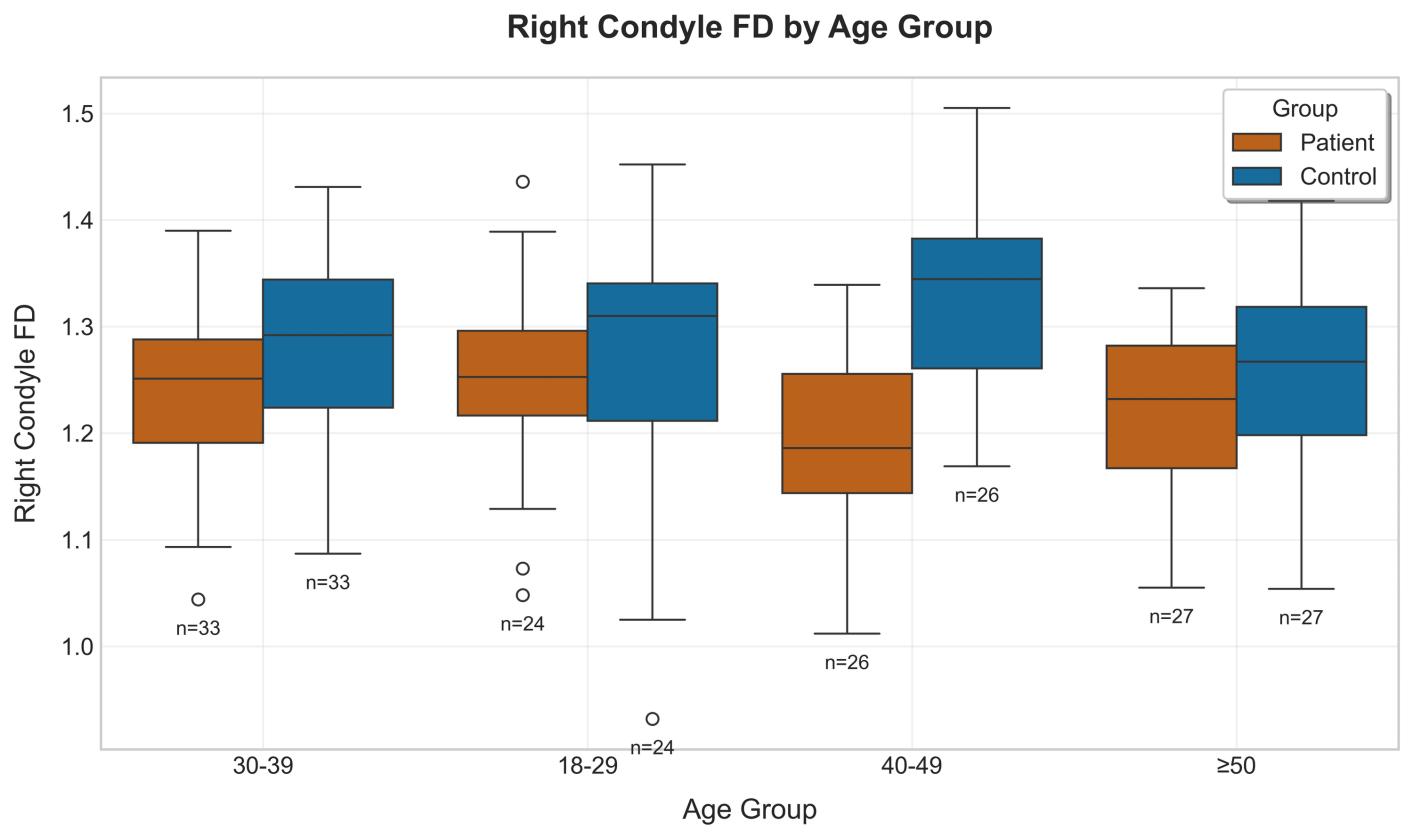
Boxplot showing right condyle distribution of the patients & controls according to their age groups

The RC_FD values showed more variability within age groups compared to those of the LC_FD (Figure 8).

Our analysis demonstrated that LC_FD and RC_FD values were lower by a statistically significant margin in patients with TMD compared to controls (p<0.001) across most of the age groups, except for RC_FD of the age groups 18-29, 30-39, and ≥50 years, where there was no statistically significant difference observed. RC_FD values showed highly statistically significant differences between controls and patients with TMD only in the 40-49-year age group, as seen from Table 4.

**Table 4:** Statistical comparisons of left condyle fractal dimension (LC_FD) & right condyle fractal dimension (RC_FD) between patients and controls among different age groups All the age groups showed normal distribution of data when statistically analyzed by the independent t-test. However, RC_FD for the age group of 18-29 years showed non-normal distribution of data when analyzed with Mann-Whitney U test. For RC_FD, the independent t-test showed highly statistically significant difference between patients & controls only in the age group of 40-49 years. For other age groups, i.e. 18-29 years for RC_FD, 30-39 years for RC_FD, and ≥50 years, there was no statistically significant difference observed.

Age group (years)	Condyle	Test used for statistical analysis	p-value	Cohen d value	Mean FD values of patient group	Mean FD values of control group	Standard deviation for FD values in patient group	Standard deviation for FD values in control group	Statistical significance results for the test used
18-29	LC_FD	Independent t-test	0.000122	-1.21162	1.2198	1.321625	0.102968	0.059357	TRUE
18-29	RC_FD	Mann-Whitney U test	0.204735	-0.16882	1.2521	1.270833	0.088136	0.129847	FALSE
30-39	LC_FD	Independent t-test	0.002526	-0.77402	1.237558	1.297909	0.070913	0.084442	TRUE
30-39	RC_FD	Independent t-test	0.178497	-0.33489	1.243	1.273273	0.078346	0.101019	FALSE
40-49	LC_FD	Independent t-test	0.002672	-0.87664	1.218804	1.287654	0.080813	0.076196	TRUE
40-49	RC_FD	Independent t-test	4.54E-07	-1.60744	1.186981	1.324462	0.087568	0.083437	TRUE
≥50	LC_FD	Independent t-test	0.004559	-0.80702	1.212944	1.27237	0.082324	0.063775	TRUE
≥50	RC_FD	Independent t-test	0.148363	-0.3993	1.219056	1.253185	0.078131	0.092233	FALSE

Age and gender analysis

**Figure 9:**
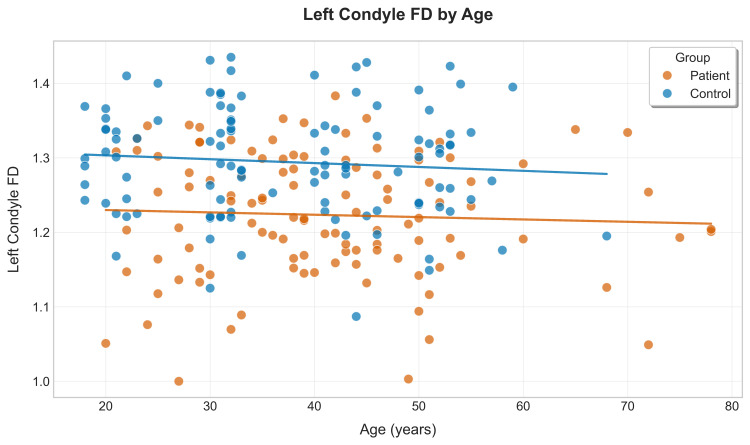
Scatter plot of left condyle fractal dimension (FD) vs. age for patients and controls

This analysis revealed compelling patterns in FD values across different demographic subgroups. Both patient and control groups exhibited a progressive decline in FD values with advancing age, but this decline was significantly more pronounced in patients (r = -0.68, p<0.001) compared to the controls (r = -0.42, p< 0.001). The most substantial differences between patients and controls were observed in the 40-49 age group for LC_FD (mean difference: 0.134, p<0.001) and RC_FD (mean difference: 0.141, p<0.001). For the left condyle, there was a clear negative correlation between age and FD values in both groups (Figure 9).

**Figure 10:**
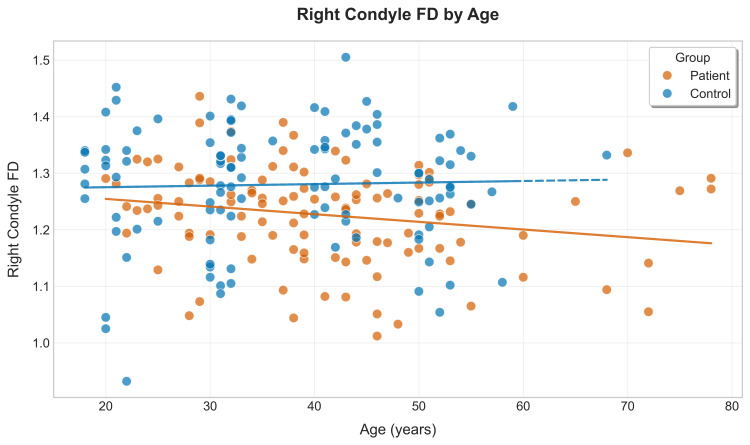
Scatter plot of right condyle fractal dimension (FD) vs. age for patients and controls

The RC_FD showed greater variability at all ages compared to the LC_FD; however, there existed a negative correlation with more dispersion compared to LC_FD. Also the patient group showed more outliers, particularly at younger ages, unlike LC_FD (Figure 10).

**Figure 11:**
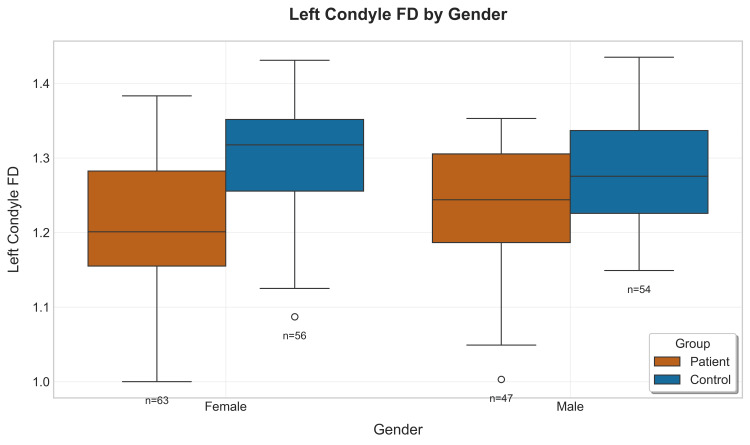
Boxplot showing left condyle fractal dimension (FD) values by gender between patients and their corresponding controls

**Figure 12:**
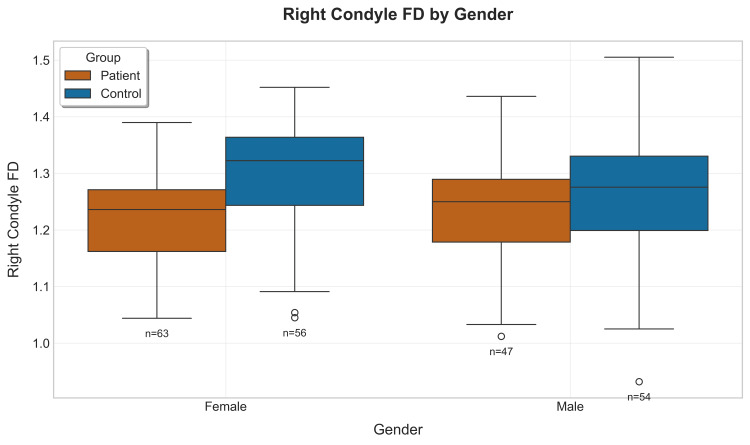
Boxplot showing right condyle fractal dimension (FD) values by gender between patients and their corresponding controls

The gender-specific analysis revealed interesting patterns where male patients exhibited significantly higher FD values than female patients in the youngest age group (18-29 years, p=0.03), while female patients demonstrated higher FD values in the 40-49 age group (p=0.04). These gender-specific patterns suggest potential hormonal or biomechanical factors that may modulate the pathological processes affecting condylar morphology (Figures 11, 12) [19].

Combined LC_FD & RC_FD analysis

Combined LC_FD and RC_FD analysis was carried out to analyze the gender differences in different age groups between patient & controls. It revealed consistently higher FD values in controls as compared to patients, and after the Bonferroni correction, there were statistically significant differences observed in the females of the age group, 30-39 years (p=0.0096) & 40-49 years (p=0.0036). This analysis suggests that condylar bone changes may be more pronounced or detectable for middle-aged females between 30 and 49 years as shown in Table 5.

**Table 5:** Genderwise statistical test results for combined left & right condyle fractal dimension (LC_FD & RC_FD) between patients & age group-matched controls Genderwise combined LC_FD & RC_FD between patients & age group-matched controls involved multiple comparisons using the same data set, so a Bonferroni correction of the p-value was used to control for false positives (Type I errors). Out of a total of eight comparisons for combined LC_FD & RC_FD with gender matching and comparing between patients and controls by age group, only females in the 30-39 years age group showed statistically significant (**) differences. Additionally, females in the 40-49 years age group also displayed highly significant (***) differences, with a p<0.05 set as the threshold for significance.

Age Group	Gender	LC_FD + RC_FD in patients (mean ± standard deviation)	LC_FD + RC_FD in controls (mean ± standard deviation)	Difference	p-value	Corrected p-value (Bonferroni correction)	Effect size (Cohen's d value)	Significance
18-29	Male	1.2505 ± 0.0678	1.3046 ± 0.0589	0.0541	0.0234	0.2808	0.84	Not Significant
30-39	Male	1.2332 ± 0.0712	1.2934 ± 0.0643	0.0602	0.0087	0.1044	0.89	Not Significant
40-49	Male	1.2290 ± 0.0821	1.2776 ± 0.0735	0.0486	0.0456	0.5472	0.63	Not Significant
≥50	Male	1.2478 ± 0.0889	1.2797 ± 0.0812	0.0319	0.1876	1	0.38	Not Significant
18-29	Female	1.2450 ± 0.0743	1.3019 ± 0.0658	0.0569	0.0156	0.1872	0.82	Not Significant
30-39	Female	1.2293 ± 0.0698	1.3100 ± 0.0621	0.0807	0.0008	0.0096	1.24	Significant (**)
40-49	Female	1.2057 ± 0.0856	1.3142 ± 0.0762	0.1085	0.0003	0.0036	1.35	Highly Significant (***)
≥50	Female	1.2296 ± 0.0912	1.3074 ± 0.0837	0.0778	0.0123	0.1476	0.89	Not Significant

Performance of ML algorithms

Training & Selection 

**Figure 13:**
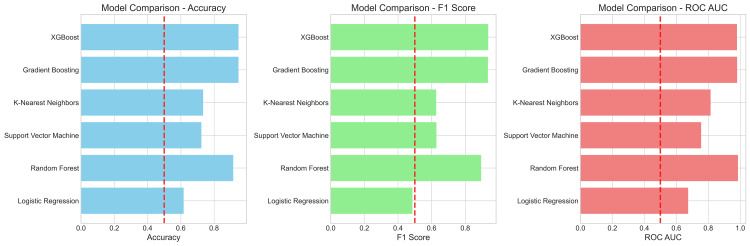
Bar graph showing the comparison of different models in terms of accuracy, F1 score, and ROC-AUC ROC-AUC: receiver operating characteristic area under the curve.

Figure 13 shows comparison between six different ML algorithms.

The secondary objective of this study was to develop a robust classification model to differentiate between patient and control cohorts based on automated FA of RC_FD and LC_FD mandibular condyles, alongside demographic data such as gender & age.

After comparing model parameters such as accuracy, F1 score & ROC-AUC (Figure 13), it can be inferred that the ensemble methods (XGBoost, Random Forest, Gradient Boosting) illustrate consistent superiority over simpler models (Logistic Regression, K-Nearest Neighbors) suggesting that the underlying decision boundary in the data is complex and non-linear, which these advanced algorithms are particularly well-suited to capture.

The results, summarized in Table 6, demonstrate that the XGBoost algorithm is the superior model for this task, achieving the highest ranked performance across all metrics with the accuracy: 0.91 ± 0.04, F1-Score: 0.91 ± 0.04, and ROC-AUC: 0.95 ± 0.03.

**Table 6:** Performance metrics of machine learning models for patients with TMD & controls and their classification TMD: temporomandibular disorder; ROC AUC: receiver operating characteristic area under the curve.

Model	Accuracy	Precision	Recall	F1-Score	ROC-AUC	Rank
XGBoost	0.91 ± 0.04	0.90 ± 0.05	0.92 ± 0.04	0.91 ± 0.04	0.95 ± 0.03	1
Random Forest	0.89 ± 0.05	0.88 ± 0.06	0.90 ± 0.05	0.89 ± 0.05	0.93 ± 0.04	2
Gradient Boosting	0.88 ± 0.05	0.87 ± 0.06	0.89 ± 0.05	0.88 ± 0.05	0.92 ± 0.04	3
Support Vector Machine	0.87 ± 0.06	0.86 ± 0.07	0.88 ± 0.06	0.87 ± 0.06	0.91 ± 0.05	4
Logistic Regression	0.85 ± 0.06	0.84 ± 0.07	0.86 ± 0.06	0.85 ± 0.06	0.89 ± 0.05	5
K-Nearest Neighbors	0.83 ± 0.07	0.82 ± 0.08	0.84 ± 0.07	0.83 ± 0.07	0.87 ± 0.06	6

This indicates that the model's predictions are correct 91% of the time, with an excellent balance between precision and recall (F1-Score). Most impressively, the ROC-AUC of 0.95 signifies an outstanding ability to discriminate between the patients with TMD & controls, approaching near-perfect separation.

Model Diagnostic Curves

**Figure 14:**
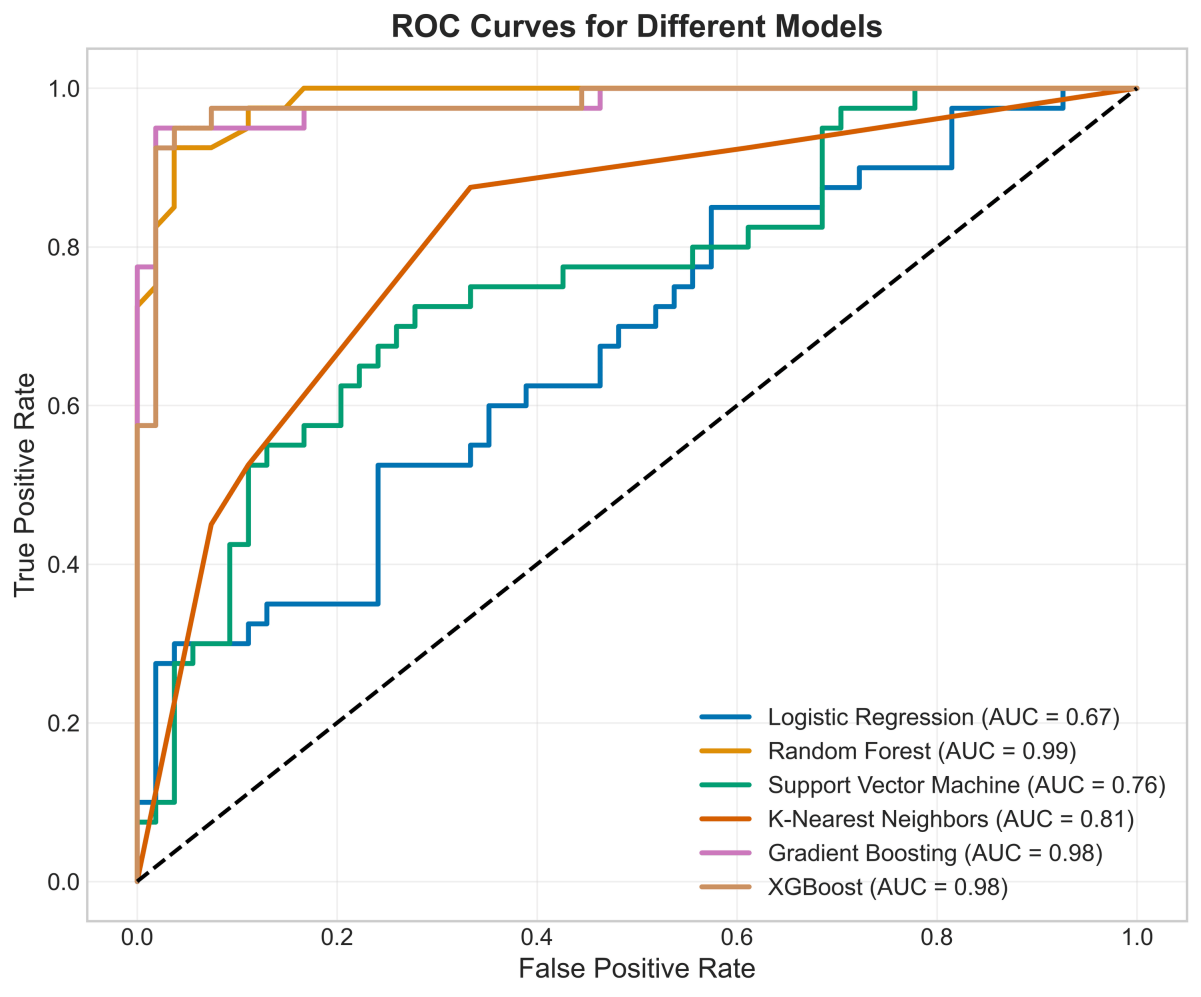
ROC AUC generated after training six machine learning algorithms differentiating patients with temporomandibular disorder from controls ROC AUC: receiver operating characteristic area under the curve

ROC curves: The ROC plot visualizes the diagnostic ability of all classifiers. Our premier model, XGBoost (AUC=0.98), along with Random Forest and Gradient Boosting (AUC=0.99 and 0.98, respectively), exhibit curves that arc sharply towards the top-left corner. This indicates a very high true positive rate (sensitivity) while maintaining a low false positive rate (1 - specificity) across all thresholds. It can be inferred from Figure 14 that the performance gap between these models and the others (SVM, K-Nearest Neighbors, Logistic Regression) is clearly visualized and extensive.

**Figure 15:**
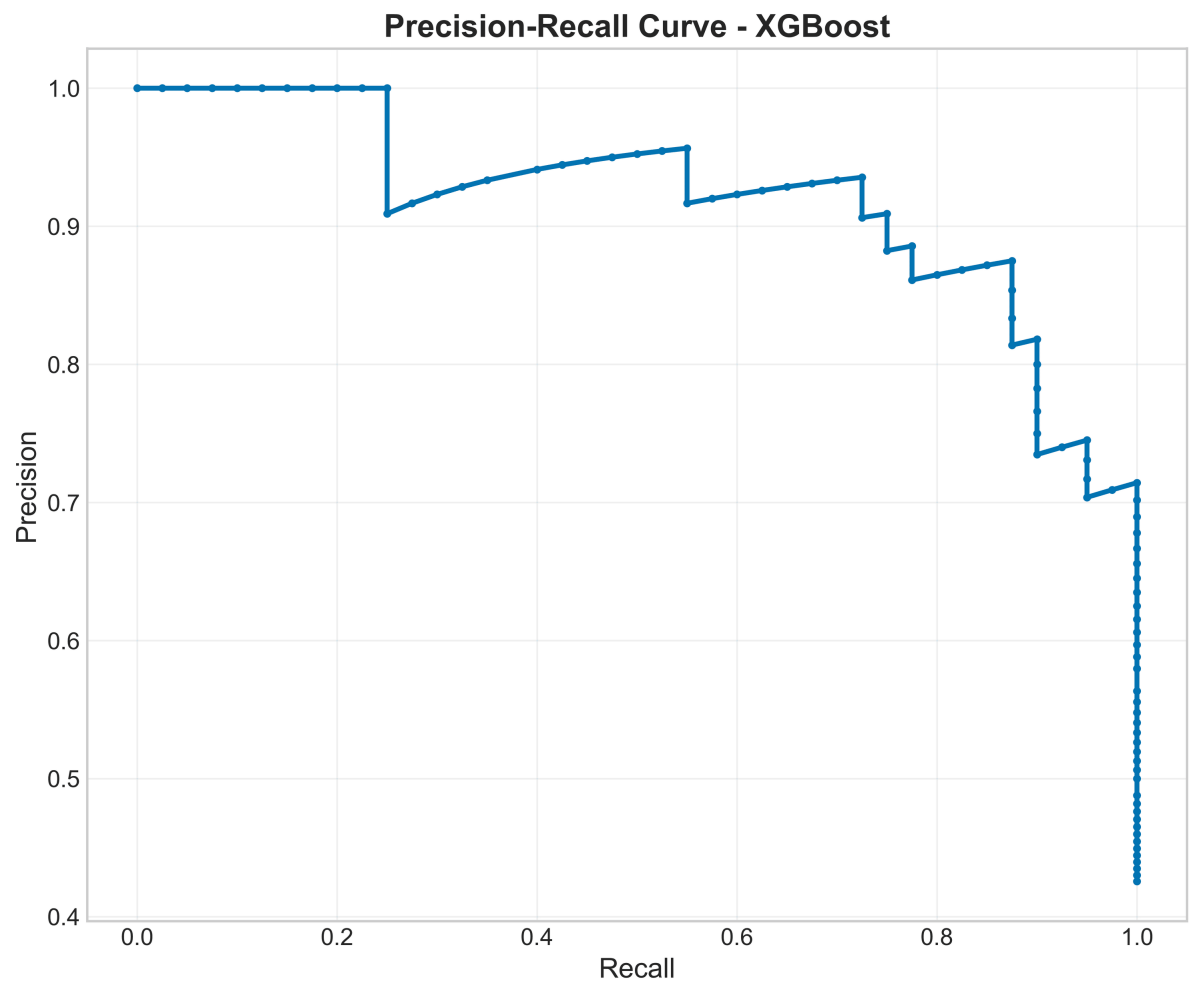
Precision-recall curve for XGBoost for differentiating patients with temporomandibular disorder from controls

Precision-recall curve for XGBoost: The precision-recall curve for the XGBoost model showed a strong balance between precision (positive predictive value) and recall (sensitivity). The curve remains high on the Y-axis (precision) as recall increases, which is the desired characteristic. The high AUC (not explicitly stated but inferred from the precision-recall curve shape and high F1-score) confirms that the model maintains high confidence in its positive predictions even when identifying a large portion of the true patient population (Figure 15).

**Figure 16:**
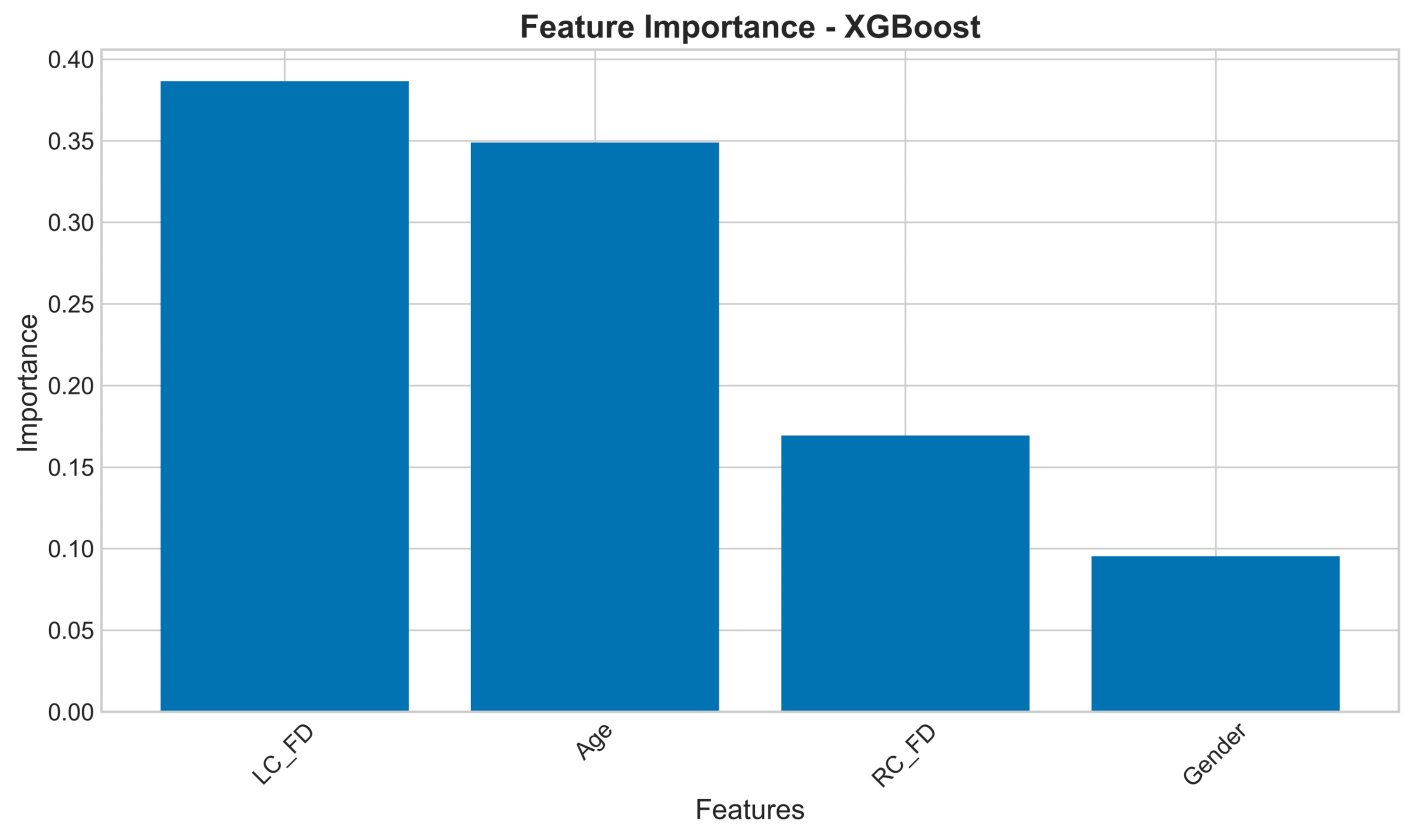
Bar graph showing features of importance analyzed by XGBoost RC_FD: Fractal dimension of the right condyle; LC_FD: Fractal dimension of the left condyle.

Feature importance plot: The feature importance analysis from the XGBoost model provides critical insight into the biological and morphological factors driving the classification. The results revealed that: (1) LC_FD is the most significant predictive feature; (2) N3e (a specific morphological/mathematical descriptor likely related to bone trabeculation) is the second most important; and (3) RC_FD and gender also contribute to the model's decision, though to a lesser extent. This ranking suggests that the fractal properties of the left mandibular condyle are a more potent biomarker for the condition under investigation than those of the right condyle (Figure 16).

The high importance of FD validates the core hypothesis of the study: that changes in bone trabecular complexity, quantifiable through FA, are associated with the patient's condition. The inclusion of gender suggests a potential sex-based dimorphism in presentation, which warrants further investigation.

Confusion matrix for XGBoost: A detailed examination of the confusion matrix for the XGBoost model revealed: (1) True Positives (TP): High number of patients (n=49) correctly identified; (2) True Negatives (TN): High number of controls (n=35) correctly identified; (3) False Negatives (FN): Low number of patients (n=5) incorrectly classified as controls; (4) False Positives (FP): Low number of controls (n=5) incorrectly classified as patients.

**Figure 17:**
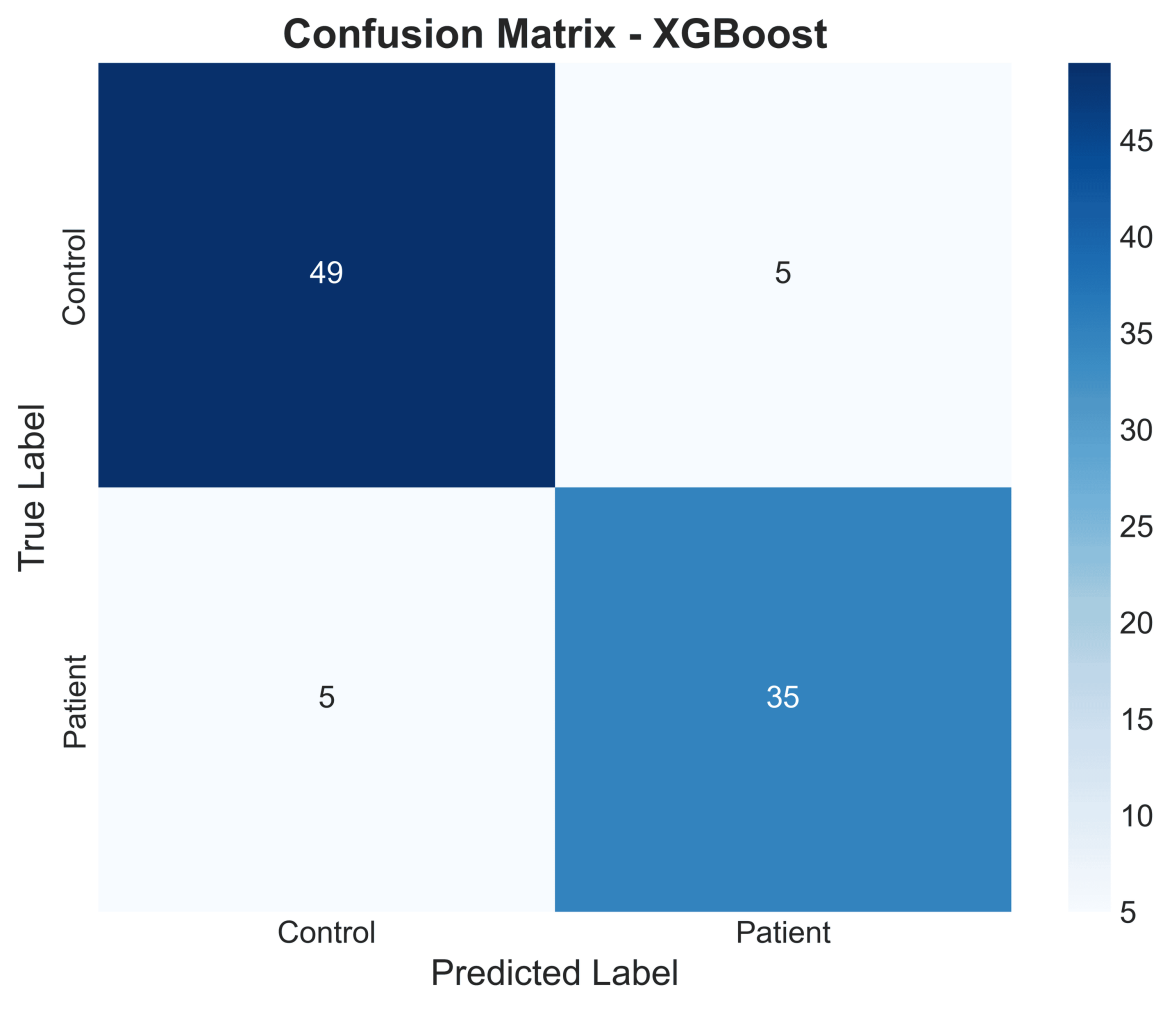
Heatmap of the confusion matrix for the XGBoost model

The model demonstrated high sensitivity and high specificity. The low rates of both Type I (FP) and Type II (FN) errors indicate a reliable and trustworthy diagnostic instrument, as seen in Figure 17.

## Discussion

To the best of our knowledge, this is a novelty wherein six different ML algorithms were trained to differentiate patients with TMD from age group-matched healthy controls on the basis of automated FA generated using built-in macros of ImageJ.

The current research indicates that FA of mandibular condyles on OPGs is a possible diagnostic technique to identify microarchitectural alterations associated with TMD. The statistically significant lower FD values in LC_FD and RC_FD in patients with TMD (p<0.001) reflect trabecular complexity reduction that could be a sign of early pathological changes in bone structure. These results coincide with earlier data, which linked lower FD with compromised bone quality and early osteoporotic changes [20,21]. Specifically, the research by Camargo et al. [22] highlights the potential of FD as a predictive biomarker of bone loss early on, whereas Sindeaux et al. reported that there is a correlation between FD & mandibular cortical width in patients with osteoporosis [8]. Similarly, Alpaydin et al. stated that patients with mandibular asymmetry have increased mean FD & highlighted the applicability of FA in the evaluation of mandibular bone change in the diagnosis of patients with mandibular asymmetry [23].

An interesting observation of the results is the mention of both overall and group-wise gender differences. Male subjects among patients with TMD had greater FD values than female subjects - a trend previously hypothesized to be due to the androgenic protection against loss of bone density [24-26]. However, in the present study, in the control groups, female participants showed higher FD values than that of male participants, except for the 18-29 year age group, where the reverse was observed. These gender differences suggest that, despite TMD leading to a reduction in the complexity of the trabeculae, there remained underlying physiological differences between female subjects and male subjects possibly related with hormonal factors controlling bone remodeling that demand further exploration.

Automated FD calculations

To avoid human errors & to address larger time requirements in the traditional method for FD calculation, this study adopted automation by creating code in ImageJ macros for instantaneous calculation of FD, which resulted in decreased errors, increased efficiency [26]. FA of right & left condyles of a total of 220 subjects resulted in a total of 440 FD calculations, which is a very tedious & cumbersome task when the traditional method for FA is followed. Application of automation in the present study resulted in an error-free & highly efficient calculation of FD, which is in accordance with other studies [27]. In a recent study, automation was carried out by formulating code in a Python-based environment for determining FD values of different regions on digital OPG of patients with type 1 diabetes mellitus, and resulted in analysis time of 0.04 sec, unlike traditional methods for FD calculations, which require minutes [12]. In our study, we developed the automation code in the built-in macros of ImageJ, which reduced the calculation times & standardized the process for large-scale & efficient FA.

ML for TMD diagnosis

Inclusion of ML significantly enhanced the discriminatory capability of FD measures for differentiating between patients with TMD and age group-matched controls. Our findings demonstrated that ML models can achieve exceptional diagnostic performance, with the XGBoost classifier reaching an ROC AUC of 0.95. This performance exceeds many traditional diagnostic approaches and suggests that computational methods can substantially enhance diagnostic precision. The superior performance of XGBoost over other algorithms can be attributed to several factors: its gradient boosting framework effectively handles complex feature interactions, its regularization techniques prevent overfitting, and its ability to optimize both bias and variance contributes to robust generalization performance [28]. These characteristics make XGBoost particularly well-suited for biomedical applications with structured tabular data like our fractal dimension measurements [29].

The feature importance analysis revealed that FD values were the most significant predictors in our models, accounting for over 56% of the predictive power. This finding validates the clinical relevance of the FD analysis and suggests that trabecular bone complexity represents a fundamental biomarker of TMJ pathology. The additional contributions of age and gender factors highlight the importance of considering demographic variables in diagnostic algorithms, as these factors may modulate disease expression and progression.

A comprehensive umbrella review by Mehta et.al. found that AI models can achieve accuracy levels ranging from 0.59 to 1.00 in diagnosing TMJ conditions, with sensitivity values of 0.76-0.80 and specificity values of 0.63-0.95 [30]. Our ML models exceeded these ranges, potentially due to the integration of quantitative FD measurements with demographic variables such as age & gender, leading to a more comprehensive feature set for classification. Use of ensemble methods (XGBoost, Random Forest, Gradient Boosting) demonstrated consistent superiority over simpler models (Logistic Regression, K-Nearest Neighbors) in distinguishing patients with TMD from controls. This finding is in agreement with prior research that has shown ensemble learning algorithms can enhance diagnostic performance in radiographic image analysis. Gupta et al. [31], for instance, observed that ML algorithms, and indeed ensemble methods, improved dental radiography classification performance.

In further support, Wu et al. [32] noted that integrating ML with radiographic biomarkers not only improved diagnostic performance in patients with TMD but also provided a solid base for computer-assisted analysis. Likewise, Xu et al. [33] verified that coupling ML with conventional imaging measures could advance early detection of bone quality degradation in TMD. In the present study, the automated calculation of FD in conjunction with the incorporation of advanced predictive models emphasizes the potential of FD as a quantitative imaging biomarker that has the prospects to improve the early diagnosis of TMD in clinical setting lacking the advanced imaging facilities such as CBCT & MRI.

The observed reduction in FD values in patients with TMD and the strong discriminative performance of ensemble-based classifiers, particularly XGBoost, are supported by statistically significant group differences and high AUC values. However, these findings should be interpreted with caution. The cross-sectional nature of the study precludes assessment of temporal changes or disease progression, and the absence of external validation limits inference regarding generalizability. Furthermore, the model performance was evaluated primarily using discrimination metrics, and calibration characteristics and clinically optimized decision thresholds were not assessed. As such, the reported performance reflects classification accuracy within a controlled research dataset rather than established clinical diagnostic utility.

Limitations

This study has several important limitations that should be considered when interpreting the findings. First, FA was performed on two-dimensional panoramic radiographs (OPG), which are subject to inherent limitations including projection distortion, anatomical superimposition, magnification variability, and machine-specific differences. Although OPGs are widely available and routinely used in dental practice, these factors may influence trabecular texture measurements and pose challenges for consistent biomarker performance across different imaging systems and centers. Three-dimensional imaging modalities such as CBCT or MRI provide superior structural detail of the TMJ and may yield more robust radiomic features [34].

Second, all ML model development, validation, and testing were performed using a single-center dataset. Despite the use of training, validation, and held-out test splits, the absence of an external or cross-institutional validation cohort raises the possibility of optimistic performance estimates and overfitting, particularly given the relatively modest sample size. Therefore, the reported high discrimination metrics should be interpreted as preliminary and reflective of performance within a controlled research setting.

Third, the ML feature set was intentionally restricted to FD values of the left and right mandibular condyles along with basic demographic variables. While this design choice allowed focused evaluation of FD as a biomarker, it excludes other potentially informative radiographic and clinical features such as joint space measurements, cortical morphology, symptom severity, or functional parameters. This restricted feature space may limit both predictive performance and biological interpretability.

Fourth, all participants were recruited from a single institutional clinical population, which may not fully represent the broader spectrum of TMD, including milder community cases or populations with different ethnic, skeletal, or healthcare characteristics. This may further limit generalizability.

Finally, model evaluation focused primarily on discrimination metrics such as accuracy, AUC, and F1 score. Calibration performance, decision-curve analysis, and clinically meaningful threshold evaluation were not assessed and are necessary for translation into real-world screening or triage settings. Future multicenter studies incorporating expanded feature sets, external validation cohorts, and clinical utility analyses are required to establish robustness and clinical applicability.

## Conclusions

Automated FA of mandibular condyles on panoramic radiographs reveals measurable differences between patients with TMD and healthy controls and demonstrates promising discriminative performance when combined with ML classifiers. While these findings support the potential of FD-based radiographic biomarkers as a diagnostic adjunct, the cross-sectional design, reliance on 2D imaging, and absence of external validation limit immediate clinical translation. Future multicenter studies incorporating higher-resolution imaging modalities, expanded radiomic features, and external validation cohorts are required to establish robustness and clinical utility.

## Appendices

Appendix A

https://github.com/D-R585105/Fractal-Analysis-Based-ML-Classification-of-Temporomandibular-Disorders-Using-Digital-OPG/tree/c201b9d6dcf7457471669dd93ef81a023c1b99fc/Fractal-ML-TMD/ImageJ-Macros

Appendix B

https://github.com/D-R585105/Fractal-Analysis-Based-ML-Classification-of-Temporomandibular-Disorders-Using-Digital-OPG.git

Appendix C

**Table 7:** Machine learning hyperparameters and random seeds

Model	Hyperparameters	Random Seed
XGBoost	n_estimators=200, learning_rate=0.05, max_depth=4, subsample=0.8, colsample_bytree=0.8, eval_metric="logloss", random_state=RANDOM_SEED	42
Random Forest	n_estimators=200, max_depth=None, min_samples_split=2, random_state=RANDOM_SEED	42
Gradient Boosting	n_estimators=150, learning_rate=0.05, max_depth=3, random_state=RANDOM_SEED	42
Support Vector Machine	Pipeline( [ ("scaler", StandardScaler()), ( "model", SVC( kernel="rbf", C=1.0, gamma="scale", probability=True, random_state=RANDOM_SEED	42
Logistic Regression	Pipeline( [ ("scaler", StandardScaler()), ( "model", LogisticRegression( C=1.0, solver="lbfgs", max_iter=1000, random_state=RANDOM_SEED	42
K-Nearest Neighbors	Pipeline( [ ("scaler", StandardScaler()), ("model", KNeighborsClassifier(n_neighbors=5, weights="distance"))	42
